# Suboptimal Intermediates Underlie Evolution of the Bicoid Homeodomain

**DOI:** 10.1093/molbev/msab051

**Published:** 2021-02-18

**Authors:** Pinar Onal, Himari Imaya Gunasinghe, Kristaley Yui Umezawa, Michael Zheng, Jia Ling, Leen Azeez, Anecine Dalmeus, Tasmima Tazin, Stephen Small

**Affiliations:** Department of Biology, New York University, New York, NY, USA

**Keywords:** gene duplication, homeodomain, TF evolution, development, Bicoid, embryo, segmentation, anterior patterning

## Abstract

Changes in regulatory networks generate materials for evolution to create phenotypic diversity. For transcription networks, multiple studies have shown that alterations in binding sites of cis-regulatory elements correlate well with the gain or loss of specific features of the body plan. Less is known about alterations in the amino acid sequences of the transcription factors (TFs) that bind these elements. Here we study the evolution of Bicoid (Bcd), a homeodomain (HD) protein that is critical for anterior embryo patterning in *Drosophila*. The ancestor of Bcd (AncBcd) emerged after a duplication of a Zerknullt (Zen)-like ancestral protein (AncZB) in a suborder of flies. AncBcd diverged from AncZB, gaining novel transcriptional and translational activities. We focus on the evolution of the HD of AncBcd, which binds to DNA and RNA, and is comprised of four subdomains: an N-terminal arm (NT) and three helices; H1, H2, and Recognition Helix (RH). Using chimeras of subdomains and gene rescue assays in *Drosophila*, we show that robust patterning activity of the Bcd HD (high frequency rescue to adulthood) is achieved only when amino acid substitutions in three separate subdomains (NT, H1, and RH) are combined. Other combinations of subdomains also yield full rescue, but with lower penetrance, suggesting alternative suboptimal activities. Our results suggest a multistep pathway for the evolution of the Bcd HD that involved intermediate HD sequences with suboptimal activities, which constrained and enabled further evolutionary changes. They also demonstrate critical epistatic forces that contribute to the robust function of a DNA-binding domain.

## Introduction

The main components of transcription networks are transcription factors (TFs) and the cis-regulatory elements of target genes that contain TF-binding sites. During evolution, DNA sequence changes in either component can alter network topology, affect gene expression patterns, and ultimately induce functional changes that are selected by evolutionary pressures (Peter and Davidson 2015). A sequence change in a cis regulatory element might affect the expression of a single gene, and it is thought that the evolution of body plan diversity is mainly driven by the accumulation of many such incremental changes (Prud’homme et al. 2007; Wray 2007; Peter and Davidson 2011). In contrast, an amino acid change that alters the DNA-binding activity of a TF would alter the expression of many target genes and cause diverse and pleiotropic effects, which might be less compatible with survival. Despite this bias, several studies in plants and animals suggest that changes in TF sequences are critical for establishing variation during evolution (Wagner and Lynch 2008; Nakagawa et al. 2013; Sayou et al. 2014).

One issue with the TF evolution hypothesis is that changes in TF function that generate new functions might interfere with critical roles normally played by the TF. However, this issue can be mitigated by gene duplication events, which provide extra genetic material for the evolution of novel or modified functions (Ohno 1970; Kondrashov et al. 2002; Singh and Hannenhalli 2008; Emerson and Thomas 2009; Vlad et al. 2014). For example, multiple duplications in the Hox locus, followed by diversification of individual genes, were critical for establishing divergent body plans throughout the metazoa (Duboule and Dollé 1989; Kappen et al. 1989; Schubert et al. 1993; Zhang and Nei 1996; Greer et al. 2000; Galant and Carroll 2002; Ronshaugen et al. 2002).

Here, we study the evolution of Bicoid (Bcd), a homeodomain (HD)-containing transcription factor that is critical for patterning anterior regions of the *Drosophila* embryo. The ancestor of *Drosophila bcd* gene (*ancbcd*) emerged ~150 Ma in Cyclorrhaphan flies (a suborder of the Diptera [two-winged flies]) after a duplication of an ancestral gene (*anczb*), which also gave rise to the ancestor of *bcd’*s sister gene *zerknullt (zen) (anczen)* (Falciani et al. 1996; Stauber et al. 1999; Schmidt-Ott et al. 2010). In *Drosophila* and most other Cyclorrhaphan flies, *anczen* maintained an ancestral role in extraembryonic patterning, whereas *ancbcd* evolved rapidly. In addition to evolution in regulatory sequences that led to maternal expression and anterior localization of *bcd* mRNA, coding sequence changes completely altered the DNA-binding activities of AncBcd (Stauber et al. 2002) and allowed it to bind to RNA (Rivera-Pomar et al. 1996; Chan and Struhl 1997). In the early embryo, Bcd protein is distributed in an anterior to posterior (AP) gradient (Driever and Nüsslein-Volhard 1988) and is essential for transcriptionally activating more than 50 genes in unique temporal and spatial patterns along the AP axis (Driever et al. 1989; Struhl et al. 1989; Chen et al. 2012). Most Bcd target genes encode transcription factors, which cross-regulate each other through space and time to form seven head segments and three thoracic segments in the anterior half of the developing embryo (Nasiadka et al. 2002). Bcd also binds directly to the mRNA of the posterior determinant *caudal* (*cad*), and prevents its translation in anterior embryonic regions (Niessing et al. 2000, 2002). Embryos lacking Bcd form no head or thoracic segments, but form posterior structures on both ends and show variable defects in abdominal segmentation (Frohnhöfer and Nüsslein-Volhard 1986).

The Zen and Bcd proteins in *Drosophila* have completely different functions in vivo. Specifically, when expressed in a Bcd-like gradient in embryos lacking Bcd, Zen has no Bcd-like activity (Liu et al. 2018). However, when the Bcd HD is swapped into the Zen protein, the chimeric ZenBcdHD partially rescues the morphological defects in Bcd-depleted embryos, and activates a subset of Bcd target genes (Liu et al. 2018). These results indicate that the unique patterning activities of Bcd are determined in large part by its DNA- and RNA-binding preferences. They suggest further that amino acid substitutions in the AncZB HD were critical for the evolution of Bcd’s functions in anterior embryo patterning.

**Fig. 1.**
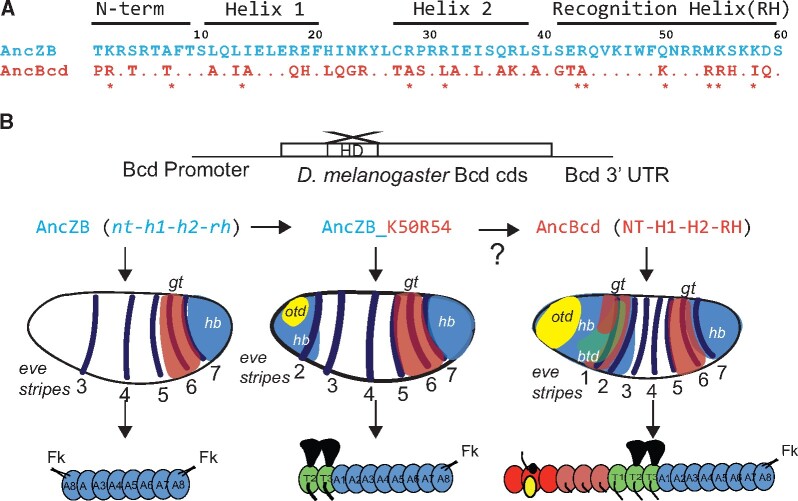
Investigating the historical amino acid changes that conferred anterior functions to AncBcd HD. (*A*) Amino acid sequences of the AncZB (blue) and AncBcd (orange) HDs (Liu et al. 2018). The four subdomains are labeled above the corresponding residues. Missing letter codes in the AncBcd sequence indicate identical resdiues with AncZB. Eleven diagnostic residues are labeled with asterisks. (*B*) Schematic of the experimental design. Chimeric HDs between AncZB and AncBcd were inserted into the coding sequence (cds) of a Bcd rescue transgene. Shown below are the results of three preliminary experiments from (Liu et al. 2018), which demonstrate that the AncZB has no rescue activity (left), the AncZB HD with a double substitution (K50R54) provides partial rescue (middle), and the AncBcd HD provides full rescue activity. The embryo schematics show expression patterns of *hunchback* (*hb*), *giant* (*gt*), *orthodenticle* (*otd*), and *even-skipped* (*eve*) in embryos with carrying rescue transgenes. In the schematics at the bottom (adapted from Lynch and Desplan 2003), blue ovals represent abdominal segments (A1–A8), green ovals represent thoracic segments (T1–T3), and red, brown, and yellow ovals represent head segments. Segments that give rise to wings and legs (T2 and T3) in the adult are shown. Filzkörper (Fk) are posterior larval structures. WebLogos generated using available Zen and Bcd HD sequences are shown in supplementary figure S1, Supplementary Material online.

**Fig. 2.**
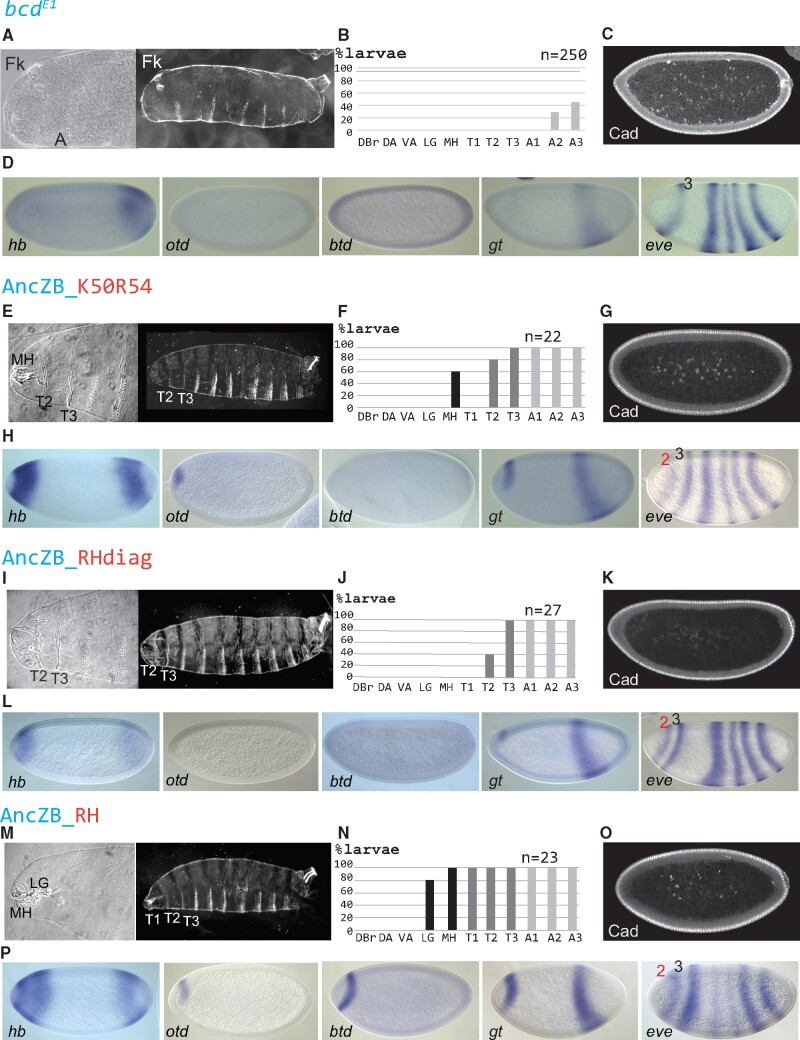
Morphological and molecular activities provided by diagnostic and nondiagnostic changes in the RH. Morphological structures and molecular activities are shown for embryos lacking Bcd (*bcd^E1^*; *A–D*), and in embryos rescued by AncZB_K50R54 (*E–H*), AncZB_RHdiag (*I–L*), and AncZB_RH (*M–P*). For each experiment, cuticle preparations of first instar anterior regions and whole larvae are shown (*A*, *E*, *I*, and *M*), along with the percentages of first instar larvae that formed specific morphological structures (*B*, *F*, *J*, and *N*). Indicated structures include Filzkörper (Fk), Dorsal bridge (DBr), Dorsal Arm (DA), ventral arm (VA), lateralgraete (LG), mouth hooks (MH), the three thoracic segments (T1–T3), and three anterior-most abdominal segments (A1–A3). (*C*, *G*, *K*, and *O*) Caudal (Cad) immunostaining in representative nc14 embryos (sagittal views). Cad protein is localized to peripheral nuclei. (*D*, *H*, *L*, and *P*) Patterns of Bcd target genes *hunchback* (*hb*), *orthodenticle* (*otd*), *buttonhead* (*btd*), *giant* (*gt*), and *even-skipped* (*eve*) in representative nc14 embryos. Measurements of anterior *hb* patterns are shown in supplementary figure S2, Supplementary Material online.

In a previous study, ancestral protein reconstruction (APR; Harms and Thornton 2010) was used to infer the amino acid sequences of the HDs that were present in AncZB and AncBcd. There are 31 high confidence differences between the two HDs, which are distributed among four HD subdomains: the N-terminal arm (NT) and three alpha helices (H1, H2, and the DNA recognition helix [RH]) (fig. 1*A*). When tested in vivo, a Bcd protein containing the AncZB HD failed to provide any Bcd-like activity, whereas an identical construct carrying the AncBcd HD completely rescued Bcd-deficient embryos to adulthood (figs. 1*B* and 2*A–D*; Liu et al. 2018). This study also tested the roles of two substitutions in the RH (q50>**K** and m54>**R**) because these had previously been shown to be critical for Bcd’s DNA-binding specificity (Treisman et al. 1989; Noyes et al. 2008) and RNA-binding activities (Niessing et al. 2000). Substituting both the K50 and R54 amino acids into the AncZB resulted in the activation of a subset of Bcd target genes, but only partially rescued the morphological defects of embryos lacking Bcd (fig. 1*B*), suggesting that other substitutions in the RH or in other subdomains of the HD were required for the evolution of AncBcd HD’s full transcriptional and posttranscriptional activities.

In this article, we present experiments designed to identify these other substitutions. We show that substitutions in the RH collectively and synergistically contribute to HD function by increasing the number of target genes regulated by the AncZB HD. However, RH substitutions alone cannot fully rescue embryos lacking Bcd to adulthood. High-frequency survival to adulthood is observed only if forward substitutions in the RH are combined with substitutions in two other subdomains (NT and H1). In contrast, combining substitutions in the RH with those in H1 or NT alone generates suboptimal HDs with lower survival rates. The distributions of larval phenotypes between these genotypes suggest different mechanisms for generating similar morphologies. Taken together, these results also suggest a multistep pathway to explain the evolutionary transition from a nonfunctional AncZB HD to an AncBcd HD with robust in vivo function through alternative pathways.

## Results

### Epistasis between Amino Acids in the RH Increased the Activity of the AncBcd HD

Among the 31 amino acid differences between the AncZB and AncBcd HDs, 11 are “diagnostic”: they are conserved in nine available fly Bcd HD sequences, and are not found in any of the 20 available insect Zen HD sequences (Liu et al. 2018; fig. 1*A* and supplementary fig. S1*A* and *B*, Supplementary Material online). Six diagnostic substitutions, including q50>**K** and m54>**R**, are present in the RH subdomain, which directly contacts base pairs in the major groove of DNA (Baird-Titus et al. 2006). We hypothesized that one or more diagnostic RH substitutions besides q50>K and m54>R might augment the degree of rescue mediated by the AncZB_**K50R54** HD (fig. 2*E*–*H*). To start, we added all four (T42, A43, R55, and I58) to the AncZB_**K50R54** HD to generate the AncZB_**RHdiag** HD (fig. 2*I*–*L*). Surprisingly, when inserted into a *bcd* rescue transgene, the AncZB_**RHdiag** HD showed a lower level of rescue than the AncZB_**K50R54** HD. For example, no head structures were observed in larvae carrying the AncZB_**RHdiag** HD, and only 40% formed two thoracic segments (compared with 80% for the AncZB_**K50R54**; fig. 2*I* and *J*, compare with fig. 2*E* and *F*). This lower level of rescue activity was also observed at the transcriptional level. Consistent with the missing head structures, expression of the head gap gene *otd* (easily detectable in embryos rescued by the AncZB_**K50R54** construct; fig. 2*H*) was not detected in embryos rescued with the AncZB_**RHdiag** construct (fig. 2*L*). We also observed reductions in the expression patterns of *hb* and *gt*, and anterior shifts of these patterns compared with those activated by the AncZB_**K50R54** HD (fig. 2*L*, compare with fig. 2*H*). For *hb*, we quantified this shift by measuring the posterior boundary position (pbp) as a percentage of embryo length (% EL, where 100%=the anterior tip, see Materials and Methods). In embryos carrying the AncZB_**RHdiag** embryos, the average position was at 82% EL (supplementary fig. S2*B*, Supplementary Material online), whereas the average pbp for the AncZB_**K50R54** construct was at 77% EL (supplementary fig. S2*A*, Supplementary Material online). Finally, the AncZB_**RHdiag** construct did not detectably repress Cad translation, neither did AncZB_**K50R54** (fig. 2*G* and *K*).

These experiments suggest that negative epistatic interactions exist among diagnostic residues in the AncZB_**RHdiag** HD, which reduce biological activity compared with the AncZB_**K50R54** double substitution. One possibility is that these negative interactions are mitigated by the other three nondiagnostic substitutions in the AncBcd RH (fig. 1*A*). To test this, we replaced the whole RH from AncZB HD with that of AncBcd (AncZB_**RH**) (fig. 2*M–P*). The addition of three more substitutions in the RH substantially improved the in vivo activity compared with both the AncZB_**K50R54** and the AncZB_**RHdiag** constructs. Around 95% of larvae containing AncZB_**RH** formed all three thoracic segments, and more than 80% formed cephalic structures (mouthhooks [MH] and lateralgraete [LG] only; fig. 2*M* and *N*). However, no larvae carrying the AncZB_**RH** construct survived to adulthood. At the molecular level, early AncZB_**RH** embryos activated transcription of the target genes *otd* and *btd* (fig. 2*P*), which were not activated by AncZB_**RHdiag** (fig. 2*L*), but failed to activate *eve* stripe 1 (fig. 2*P*). Also, the expressions of *hb* and *gt* were stronger in AncZB_**RH** embryos compared with AncZB_**RHdiag**. In particular, the average *hb* pbp in AncZB_**RH** embryos was at 73% EL (supplementary fig. S2*C*, Supplementary Material online), which is more posteriorly localized compared with 82% EL in AncZB_**RHdiag** embryos (supplementary fig. S2*B*, Supplementary Material online). However, we could not detect any significant repression of Cad translation in AncZB_**RH** embryos (fig. 2*O*).

Taken together, these results suggest that positive and negative epistatic interactions within the RH were critical for the evolution of the AncBcd HD. However, none of the RH substitutions tested here mediate full rescue of Bcd-deficient embryos to adulthood, indicating that additional substitutions in other subdomains were required for the acquisition of Bcd’s novel patterning activities.

### Combining Substitutions in Three Subdomains Were Required for the Evolution of Robust AncBcd HD Function

**Fig. 3.**
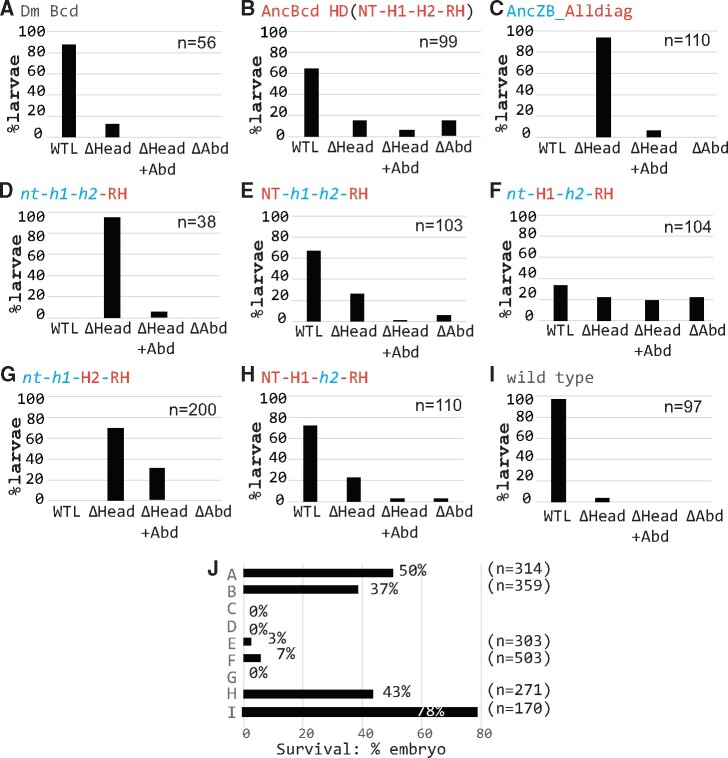
Different phenotypes observed among *Drosophila* larvae expressing ancestral HD proteins. (*A–I*) Percentages of Bcd-deficient first instar larvae rescued with the indicated transgenes and wild-type larvae cuticle that appear wild-type like (WTL), or have head defects (ΔHead), head plus abdominal defects (ΔHead+Abd), or abdominal defects alone (ΔAbd). (*J*) Survival rates of embryos to adulthood in the corresponding transgenic and wild-type lines. See supplementary figure S3, Supplementary Material online, for images of representative larvae in each phenotypic category.

We tested several different constructs that combine forward substitutions in the RH with those in other subdomains. In these experiments, the cuticle patterns of first instar larvae containing each construct were highly variable, so we divided them into the following four categories (see Materials and Methods): 1) WTL (wild-type like): larvae with easily detectable head structures (MH, LG, VA, DA, and DBr), three thoracic segments, and eight abdominal segments. 2) ΔHead: larvae missing any of the five head structures mentioned above. 3) ΔHead+Abdomen: larvae with head defects and additional defects in abdominal segments. 4) ΔAbdomen: larvae with normal head structures, but with defects in abdominal segments. As positive controls, we assayed the rescue activities of transgenes containing the wild-type *Drosophila* Bcd HD and the reconstructed AncBcd HD, which produced ∼90% and ∼65% WTL larvae, respectively (fig. 3*A* and *B*; supplementary fig. S3*A* and *B*, Supplementary Material online). For the wild-type transgene, the remaining 10% were classified as ΔHead, and no larvae showed abdominal defects. In contrast, larvae rescued with the AncBcd HD that were not classified as WTL showed more variability, with around 20% with head defects alone or a combination of head and abdominal defects. An additional 15% contained well-formed head structures, and defects in abdominal segments, which ranged from a mild phenotype missing some abdominal segments to a strong phenotype lacking all abdominal segments and poorly formed filzkörper (supplementary fig. S3*B*, Supplementary Material online).

**Fig. 4.**
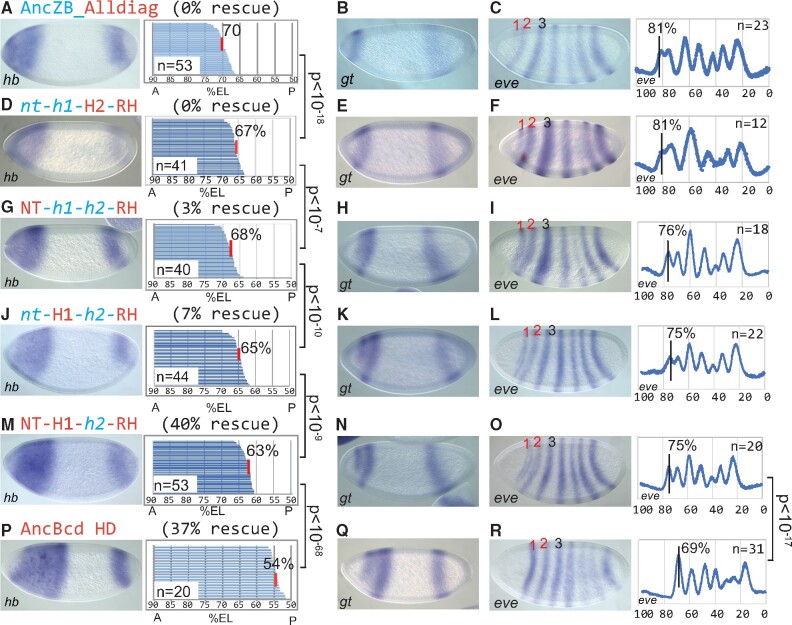
*hb*, *gt*, and *eve* expression patterns in lines exhibiting different levels of phenotypic rescue. Tested HDs are labeled as in figure 3. (*A*, *D*, *G*, *J*, *M*, and *P*) Representative nc14 embryos stained by *in situ* hybridization to detect *hb*. Panels show the *hb* posterior boundary position (pbp) (% EL; anterior tip=100%) of embryos in each group. Each horizontal line in each panel represents the anterior *hb* expression pattern in a single embryo, and the average pbp is denoted by a vertical red line. *P* values between corresponding lines were calculated using Student’s *t*-test. (*B*, *E*, *H*, *K*, *N*, and *Q*) Representative nc14 embryos stained for *gt* expression. (*C*, *F*, *I*, *L*, *O*, and *R*) Representative nc14 embryos stained for *eve* expression. *eve* stripe 1 positions were calculated from more than ten individual embryos and are denoted by vertical black lines. The *P* value of mean difference of *eve* stripe 1 positions between AncBcd and [CH(NT-H1-*h2*-RH)] and optimal vs. suboptimal rescue, respectively, was calculated using Student’s *t*-test. The distribution of *eve stripe* 1 positions in rescuing vs. nonrescuing HD chimeras are shown in supplementary figure S5, Supplementary Material online.

As mentioned above, the ancestral reconstructions of the AncZB and AncBcd HDs identified 11 diagnostic changes distributed across all four subdomains (fig. 1*A*). We hypothesized that forward substitutions at all 11 diagnostic positions might convert the inactive AncZB HD into a fully active HD. Thus, we made all 11 substitutions in the AncZB HD (AncZB_**Alldiag**), and tested the construct for rescue activity. These substitutions in multiple subdomains showed partial patterning activity, with more than 90% of larvae forming all three thoracic segments and at least one of the head structures mentioned above (supplementary fig. 3*D*, Supplementary Material online). At the molecular level, the AncZB **Alldiag** construct activated all tested Bcd target genes, though weaker (fig. 4*A* and supplementary fig. S4*A*, Supplementary material online). In addition, this construct also consistently suppressed translation of Cad in anterior regions (supplementary fig. S4*A*, Supplementary material online). However, no larvae rescued by the AncZB_**Alldiag** transgene formed all five assayed head structures, so none could be classified as WTL (fig. 3*C*), and no larvae survived to adulthood (fig. 3*J*).

We next tested the rescue activity of three chimeric HDs that separately combine all forward substitutions (diagnostic and nondiagnostic) in the RH with those in each of the other subdomains [CH(**NT**-*h1*-*h2*-**RH**), CH(*nt*-**H1**-*h2*-**RH**), and CH(*nt*-*h1*-**H2**-**RH**)]. Transgenes containing two of these chimeric HDs strongly increased rescue activity compared with the transgene containing all changes in the RH alone (AncZB_**RH**) (fig. 3*E* and *F*, compare with fig. 3*D*, also supplementary fig. S3*E* and *F* compare with supplementary fig. S3*C*, Supplementary Material online). Nearly 70% of Bcd-deficient larvae carrying the CH(**NT**-*h1*-*h2*-**RH**) transgene were classified as WTL (fig. 3*E*), but in many cases, specific head structures, including the lateralgraete and the dorsal and ventral arms, appeared shorter than normal (supplementary fig S3*E*, Supplementary Material online). An additional 25% failed to form one or more head structures. For the CH(*nt*-**H1**-*h2*-**RH**) transgene, there was also a strong increase in rescue activity, but less than 40% were classified as WTL, with the rest evenly distributed among the other three phenotypic categories (fig. 3*F* and supplementary fig. S3*F*, Supplementary Material online). In contrast, no WTL larvae were produced by the CH(*nt*-*h1*-**H2**-**RH**) rescue transgene (fig. 3*G* and supplementary fig. S3*G*, Supplementary Material online).

We performed hatching tests (see Materials and Methods) to monitor the frequency of survival past larval stages for the experiments that produced WTL larvae (fig. 3*J*). As a baseline, the frequency of survival to adulthood for wild-type larvae under our laboratory conditions was ∼80% (fig. 3*I* and *J*). In contrast, the positive control transgenes containing endogenous Bcd and AncBcd HDs resulted in the survival of only 50% and 37% of larvae to adulthood respectively (fig. 3*J*), perhaps due to the fact that the transgenes are inserted into an ectopic genomic position. Remarkably, both chimeric constructs that yielded WTL larvae [CH(**NT**-*h1-h2*-**RH**) and CH(*nt*-**H1**-*h2*-**RH**)] directed the survival of 3% and 7% of those larvae to adulthood, respectively (fig. 3*J*). Although these survival frequencies are quite low compared with the control experiments, they show that the full developmental function of the Bcd HD can be achieved by substitutions in two different combinations of subdomains (NT+RH and H1+RH).

We also tested if combining substitutions in the NT, H1, and RH subdomains would increase the rate of survival to adulthood. Around 60% of larvae produced by *bcd* females containing the CH(**NT**-**H1**-*h2*-**RH**) were classified as WTL (fig. 3*H* and supplementary fig. S3*H*, Supplementary Material online), and 43% survived to adulthood (fig. 3*J*). This result shows that combining substitutions in three separate subdomains is required and sufficient for generating a Bcd HD with high penetrance rescue activity.

### Bcd Target Gene Positions That Correlate with Full Rescue to Adulthood

To understand the molecular basis for the differential rescue mediated by the chimeric HDs, we examined the expression patterns of several Bcd target genes, starting with Cad, which is translationally suppressed by Bcd in wild-type embryos. As expected, all three chimeric HDs that direct full rescue also show Cad suppression (supplementary fig. S4*C*–*F*, Supplementary Material online). In contrast, most constructs that failed to rescue to adulthood also failed to detectably suppress Cad (fig. 2 and supplementary fig. S4*B*, Supplementary Material online). However, the construct containing the AncZB_**AllDiag** HD, which failed to rescue to adulthood (fig. 3*J*) did suppress Cad (supplementary fig. S4*A*, Supplementary Material online). Previous studies have shown that Cad suppression is not absolutely required for embryo survival but it might be necessary under stress as shown by the temperature-sensitive head defects in Cad RNA-binding mutant Bcd larvae (Niessing et al. 2000). Taken together, these results show that suppression of Cad may be required for robust rescue to adulthood, but it is not sufficient.

We next examined *hb* expression in embryos containing the chimeric HD transgenes (fig. 4*A*, *D*, *G*, *J*, *M*, and *P*). Our experiments with HDs containing RH substitutions alone showed that the degree of partial rescue activity is positively correlated with the extension of the *hb* expression domain into middle regions of the embryo (supplementary fig. S2, Supplementary Material online). In addition, the *hb* posterior boundary position (pbp) in embryos carrying the AncZB_**Alldiag** construct was located at 70% EL, relatively far from the boundary position in embryos rescued by the AncBcd HD (54% EL) (fig. 4*A*, compare with fig. 4*P*). Thus, we hypothesized that HDs capable of directing full rescue to adulthood might activate *hb* domains that extend farther posteriorly than those that fail to rescue. Indeed, embryos containing all three fully rescuing constructs show *hb* posterior boundary positions (pbps) that range from 68% to 63% EL (fig. 4*G*, *J*, and *M*). However, the correlation between *hb* boundary positioning and full rescue is not perfect. Specifically, the CH(*nt-h1*-**H2-RH**) chimera, which completely failed to fully rescue (fig. 3*J*), activated a *hb* domain with a pbp at 67% EL (fig. 4*D*). Therefore, these results suggest that extending the *hb* domain to a specific AP position is also required, but not sufficient for the mediating the full regulatory activity of the AncBcd HD.

We also examined the expression of the gap genes *otd*, *btd*, *gt*, and the pair-rule gene *eve* (supplementary fig. S4, Supplementary Material online and fig. 4). There were no detectable differences in the expression patterns of *otd* and *btd* between embryos carrying the three chimeric transgenes that fully rescue and those that do not (supplementary fig. S4, Supplementary Material online). In contrast, the anterior expression pattern of *gt* showed significant differences. The anterior *gt* domain initially appears as a broad stripe, which resolves over time into two stripes (Mohler et al. 1989; Eldon and Pirrotta 1991; Kraut and Levine 1991). The separation into stripes occurs in all three lines that direct full rescue (fig. 4*H*, *K*, and *N*), but not in embryos carrying the CH(*nt-h1*-**H2**-**RH**) chimera (fig. 4*E*), or in any other tested constructs that fail to direct full rescue (figs. 2*H*, *L*, and *P* and 4*B*). We also observed a strong correlation between fully and partially rescuing chimeric lines and the positioning of *eve* stripe 1. Embryos carrying the three transgenes that fully rescue formed *eve* stripe 1 at 75–76% EL (fig. 4*I*, *L*, and *O*), whereas the CH(*nt*-*h1*-**H2**-**RH**) and AncZB_**Alldiag** transgenes (both 0% full rescue) consistently formed this stripe more anteriorly (81% EL; fig. 4*C* and *F*; supplementary fig. S5, Supplementary Material online). Also, *eve* 1 was more clearly separated from *eve* 2 in embryos that fully rescue to adulthood. Thus, there is a perfect correlation between the ability to fully rescue to adulthood, the separation of the anterior *gt* domain into two stripes, and the positioning of *eve* 1. However, the positions of the *gt* domain and *eve* 1 even in these fully rescuing lines were still significantly anterior compared with the control AncBcd HD line (fig. 4*H*, *K*, and *N*, compare with fig. 4*Q*;fig. 4*I*, *L*, and *O*, compare with fig. 4*R*).

Although we observed substantial differences in *gt* and *eve* patterning between fully and partially rescuing lines, we detected only one slight expression difference that might explain the different survival rates (3–40%) among the three constructs that fully rescue. Notably, the CH**(NT-H1**-*h2*-**RH**) transgene, which combines forward substitutions in three subdomains and rescues 40% of *bcd* mutant embryos to adulthood activated *hb* expression with a pbp at 63% (fig. 4*M*). This position is slightly posterior compared with the boundaries in embryos rescued by the CH(**NT-***h1*-*h2*-**RH**) or CH(*nt***-H1**-*h2*-**RH**) transgenes (68% and 65%, respectively, fig. 4*G* and *J*). Aside from this difference, we detected no significant changes in any of the tested gap gene or *eve* expression patterns among these constructs.

## Discussion

### Molecular Requirements for the Patterning Activity of the AncBcd HD in *Drosophila*

In this article, we used an in vivo *Drosophila* rescue assay to study the impact of the historical coding sequence changes on the evolution of Bcd HD’s developmental functions. By making chimeric HDs between the AncZB (no function) and the AncBcd (full function) HDs, we showed that the substitutions in at least three separate subdomains (NT, H1, and RH) must be combined for full patterning activity.

AncBcd evolved to suppress translation of Cad and activate transcription of a large number of target genes at different positions along the AP axis of the embryo. Our results shed light on the molecular requirements for both of these activities. The R54 residue in Bcd was previously shown to be required for Cad suppression (Niessing et al. 2000), but our data suggest that it is not sufficient, even in combination with the other eight forward substitutions in the RH of AncBcd (AncZB_**RH**). However, by combining the RH substitutions with several different sets of substitution in the NT and/or H1 or substituting all diagnostic residues across all subdomains, variable levels of suppression were achieved (supplementary fig. S4, Supplementary Material online). The impact of the level of suppression on rescue potential and patterning is not known, and will be addressed by future experiments.

At the transcriptional level, it was previously shown that inserting K50 alone into the AncZB HD caused the activation of only three of eight tested target gene responses, whereas the double substitution (K50R54) increased that number to five (Liu et al. 2018). In this article, we show that substituting all nine amino acids from the RH of AncBcd HD into AncZB resulted in the activation of all tested target genes except *eve stripe* 1 and the splitting of the anterior domain of *gt* into two stripes (fig. 2*P*). Moreover, all activated Bcd-dependent expression patterns were anteriorly shifted. Combining substitutions in the RH with those in NT and/or H1 had major effects on the gene expression patterns: they led to the activation of *eve* stripe 1, and extended or shifted critical expression patterns into more posterior positions, which might have allowed for splitting of the anterior *gt* domain.

The correlation between target gene expansion and rescue activity is most easily observed for the target gene *hb*, which encodes a critical cofactor for activation of all Bcd-dependent target genes (Simpson-Brose et al. 1994; Ochoa-Espinosa et al. 2005; Porcher and Dostatni 2010; Schroeder et al. 2011), and functions as an important repressor to prevent posterior gap gene expression in anterior regions of the embryo (Hülskamp et al. 1990; Struhl et al. 1992; Wu et al. 2001; Yu and Small 2008). In embryos carrying constructs that fail to fully rescue to adulthood, *hb* pbps are located between 82% and 67% EL, whereas embryos carrying constructs with full rescue activity form *hb* pbps at the posterior limit of this range (68% EL) or farther posterior. Interestingly, the **CH**(**NT-H1**-*h2*-**RH)** construct, which rescues to adulthood with a frequency similar to that observed for the AncBcd HD control, forms a *hb* pbp at 63%. We propose that the position of ∼65% EL establishes the minimal amount of embryonic space required for the correct placement of gap and pair-rule stripes, robust formation of cephalic structures, and ultimately survival to adulthood. The pbp at 65% EL is significantly more anterior than those directed by the control AncBcd construct (54% EL, fig. 4*P*) or wild-type embryos (54%; Chen et al. 2012), but is very close to the *hb* pbp in embryos laid by heterozygous *bcd* females (∼61% EL), which survive with high penetrance (Liu et al. 2013). How interactions between the RH and other HD subdomains cause posterior extensions of the zygotic *hb* domain is not clear; they could indirectly modify the DNA-binding preferences of the HD or mediate interactions with maternal cofactors such as Hb or Zelda, both of which are critical for Bcd’s in vivo functions in *Drosophila* (Simpson-Brose et al. 1994; Porcher and Dostatni 2010; Xu et al. 2014; Hannon et al. 2017; Mir et al. 2017; Datta et al. 2018).

### Evolution of the AncBcd HD through Suboptimal Intermediate Steps

An ancient duplication of AncZB led to the evolution of the K50 HD protein Bcd as a key regulator of anterior development in the Cyclorrhaphan suborder of the Diptera (Stauber et al. 1999). No other suborders of the Diptera or other insects contain Bcd; in these insects, maternal Bcd’s roles in anterior patterning must be fulfilled by other gene(s). In the jewel wasp *Nasonia*, Bcd-like activity is provided by maternal Orthodenticle (Otd), another K50 HD protein. Unlike Bcd, Otd is highly conserved, and because it binds in vitro to DNA sequences similar to those bound by Bcd, it has been proposed that Bcd evolved to take over regulation of an ancestral Otd-dependent network (Lynch and Desplan 2003). In *Drosophila*, *otd* became a Bcd target gene, and has maintained a critical role in the specification of head segments (Gao and Finkelstein 1998; Datta et al. 2018).

Our data show that changes in the AncZB HD changed its DNA and RNA binding activities, and allowed it to bind RNA, gain new target genes, and acquire novel roles in patterning thoracic and abdominal segments. Importantly, the evolution of Bcd occurred specifically in the Cyclorrhaphan lineage. In other species, proteins unrelated to Bcd and Otd (e.g., a homolog of Odd-paired in the drain fly *Clogmia*, and a cysteine clamp protein in the midge *Chironomus*) have been proposed as important maternal factors involved in anterior embryo patterning (Klomp et al. 2015; Yoon et al. 2019). Together these studies suggest that the earliest events of embryo patterning are dynamically changing during the process of evolution.

**Fig. 5.**
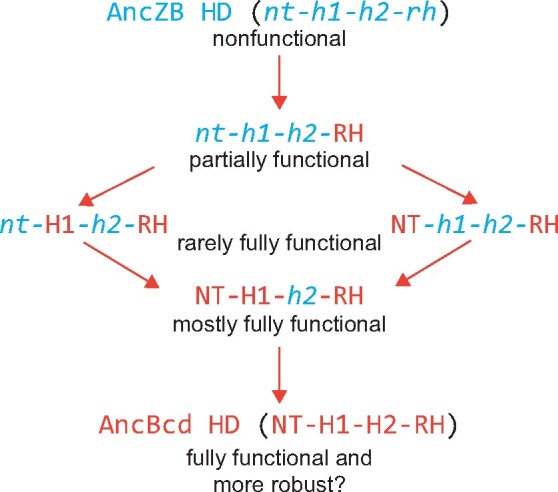
A proposed multistep pathway for the evolution of the AncBcd HD. Orange arrows represent amino acid substitutions in individual subdomains. In the first step, initial substitutions in the RH changed the DNA-binding preferences of the HD, and allowed it to bind to RNA. In a second step, these initial substitutions were followed by additional changes in either the NT or the H1 subdomain, each of which could have significantly augmented the in vivo activities of the evolving HD in a small percentage of embryos. In a third step, substitutions in the unchanged subdomain (H1 for RH+NT or NT for RH+H1) would further increase patterning activity and raise the survival rate to almost control levels.

Our data show that robust patterning function of the AncBcd from AncZB is achieved by combining forward substitutions in three subdomains (RH, NT, and H1). It seems impossible that critical amino acid substitutions in all three subdomains occurred simultaneously at some point in the evolution of the AncBcd HD. However, we propose that critical substitutions in each of the three might have occurred in a specific temporal order, each of which endowed the protein with a novel property that could be positively selected in evolving flies (fig. 5).

Assuming that the ancestral network was controlled by a K50 HD protein such as Otd (Lynch and Desplan 2003), we propose that the first step involved multiple substitutions in the RH, including q50>K and m54>R. The codons for Q (Gln: CAA and CAG) and K (Lys: AAA and AAG) differ by only one base, so the q50>K transition involved only a single base-pair substitution that would have dramatically changed the evolving protein’s DNA-binding preference. Reverse substituting or mutating K50 completely abolishes AncBcd HD function (Liu et al. 2018), which means that the effects of all other substitutions in the evolving HD were dependent on keeping the K50 residue intact. If this substitution occurred in an ancestral fly with an Otd-dependent anterior patterning network, the evolving protein would be immediately available to bind to many Otd-dependent target genes, which might have provided a selective advantage. The m54>R substitution, which also involves a single base change (AUG to AGG), might have refined DNA-binding specificity to increase the number of activated target genes, and set the stage for other substitutions that allowed the AncBcd HD to bind to RNA. K50 and R54 are present together only in Bcd HDs (Noyes et al. 2008), consistent with the possibility that this combination might have been under positive selection. In addition to the q50>K and m54>R substitutions, there are seven other amino acid differences between the RH subdomains of AncZB and AncBcd. It is not clear which of these are required for AncBcd HD function, or when they appeared historically. However, one combination of six substitutions tested here (AncZB_**RHdiag**) reduced HD activity compared with the K50R54 double substitution. This result suggests that interactions between amino acids constrained the historical order of substitutions in the RH subdomain.

Although robust HD activity requires substitutions in three subdomains, forward substitutions in either NT or H1 substantially augment the rescue activity generated by changes in the RH alone. Specifically, AncZB HDs containing either combination (RH+NT or RH+H1) rescue a small percentage of embryos that survive to adulthood (fig. 3*J*). We propose that the addition of substitutions in either NT or H1 represent alternative second steps in the historical evolution of AncZB HD (fig. 5). Either combination (RH+NT or RH+H1) would have generated a suboptimal intermediate HD configuration that could have been positively selected for and stabilized, perhaps by increasing the fitness of a subpopulation in specific physical/environmental conditions. Once stabilized, in a third step, substitutions in the other critical subdomain (H1 for the NT+RH intermediate, for example) would further increase HD activity and robustness of the evolving HD.

### Limitations and Challenges for the Future

Our results shed light on the mechanisms involved in the evolution of the AncBcd HD, but are limited by the fact that all chimeric HDs were inserted into the modern-day *Drosophila* Bcd protein. As such, these experiments do not take into account the evolution of other parts of the protein, which show even greater levels of amino acid sequence divergence. Further, all our experiments were performed in modern-day *Drosophila* embryos, and do not take into account changes in the cis-regulatory elements of target genes that coevolved with the AncBcd protein. However, as the genome sequences of more insects become available, it should be possible to use reconstruction strategies to define the ancestral sequences of the complete AncBcd protein and the regulatory regions it interacts with. Furthermore, the ever-increasing use of CRISPR/Cas9 techniques for gene editing in nonmodel organisms should allow for testing ancestral protein and regulatory sequences in multiple insect species. Although these methods cannot create the ancestral systems themselves, they should make it possible to discover general features that permit a transcription factor and its target regulatory sequences to coevolve.

## Materials and Methods

### Drosophila Stocks, Cloning, and Transgenesis

We used the following stocks from our own lab for these experiments: yw (wild type), *Cyo bcd*^+^/*Sco*; *bcd^E1^*/*bcd^E1^*, yw; TM3B, Sb, Ser/D and ΦC31 (y+); 38F1 (w+). We cloned an injection plasmid (piattB40-Bcd) containing two inverted ΦC31-specific recombination sequences, a Gmr-GFP reporter, and a polylinker flanked by 1.9-kb *bcd* promoter and 0.8-kb 3′-UTR. The *bcd* coding region was amplified by PCR from pBS-SK+ cDNA clones, digested with RsrII and AscI and ligated into piattB40-Bcd in between Bcd promoter and 3′-UTR. This main plasmid was used to generate Dm Bcd protein with different ancestral HDs, which are predicted as described and published in (Liu et al. 2018). We used standard cloning techniques to generate homeodomain swaps and residue changes. Gene Blocks coding for the ancestral and chimeric HD sequences together with the flanking Bcd coding sequence were obtained from Integrated DNA Technologies (IDT). They were digested with AscI and BspEI and ligated to the piattB40-Bcd vector digested with the same restriction enzymes. The cloned sequences were confirmed by Sanger sequencing before and after transgene insertion. All transgenic lines were generated using the ΦC31 integration system (Recombination mediated cassette exchange, RMCE), and constructs were integrated into the 38F1 landing site on the second chromosome (Bateman et al. 2006). Each transgene was crossed to *Cyo bcd*^+^/*Sco*; *bcd^E1^*/*bcd^E1^* to generate *Cyo bcd*^+^/*[transgene]*; *bcd^E1^*/*bcd^E1^* stocks*.* Embryos and larvae from homozygous transgenic females were assayed for gene expression and cuticle phenotype.

### Generating Sequence Logos

The logo generation platform (http://weblogo.berkeley.edu/logo.cgi) (Crooks et al. 2004) was used to process multiple protein sequence alignments provided in FASTA (Pearson and Lipman 1988) format and to generate the logos for the insect homologs of 20 Zen and nine Bcd HD sequences. The analyses to predict the simple ancestral sequences shown in figure 1*A* were explained in detail in our previous work (Liu et al. 2018). The codes for all the analysis are freely available. Briefly, 33 curated HD sequences from 27 insect and arthropod species were used to reconstruct the ancestral sequences on a tree topology a priori to constrain all species relationships, which are well corroborated from extensive prior research on insect phylogenetics. Branch lengths and model parameters were then optimized on this tree by maximum likelihood. The results were ambiguous in three locations. The alternatives at these locations were tested in vitro with gel shift assays to show their similar functions (Liu et al. 2018).

### In Situ Hybridization, Immunohistochemistry, and Image Processing

*In situ* hybridizations were performed as previously described (Small 2000). Briefly, embryos 1–3 h AEL (after egg laying) were dechorionated 2 min in 100% bleach, fixed, and devitellinized in a biphasic fixation solution containing 3 ml 1× PBS, 1 ml 37% formaldehyde, and 4 ml heptane for 25 min on a shaker at RT. Fixed and permeabilized embryos were incubated with DIG or fluorescein-labeled RNA probes and the labeled probes were detected by Alkaline Phosphatase (AP)-conjugated primary antibodies (Roche Cat No. 11093274910, RRID: AB_514497 and Roche Cat No. 11426338910, RRID: AB_514504) by using NBT/BCIP solution (Roche Cat No. 19315121). RNA expression was observed by Zeiss Axioskop microscopy.

Guinea pig anti-Cad (Kosman et al. 1998) (1:400) and Alexa Fluor conjugated 647 donkey anti-guinea pig (1:500) (Molecular Probes Cat No. A-21447, RRID: AB_141844) were used to examine Cad protein expression. All antibodies were diluted in PBT (1× PBS with 0.1% Tween). Data for immunostaining images were collected on a Leica TCS SP8 confocal microscope using the Leica confocal analysis software.

### Larval Phenotype Analyses and Hatching Assays

Cuticle preparations were performed on embryos aged 24–30 h at 25 °C as previously described (Wu et al. 1998). Briefly, larvae were dechorionated for 2 min in 100% bleach, and a 1:1 mixture of methanol and heptane was used to remove the vitelline membrane and fix the larvae. Then these larvae were mounted in 1:1 mixture of Hoyer’s medium (Anderson 1954) and lactic acid and incubated o/n at 65 °C to digest inner tissues.

Dark-field views of whole larvae were imaged at 200× magnification; DIC images of cephalic regions are imaged at 400×. Each image was sorted into one of four categories that encompassed the variation in phenotype of first instar larvae both within each transgenic fly line, and across all fly lines analyzed. The different phenotypic categories are; WTL: larvae containing all head segments (MH, LG, VA, DA, and DBr [Dorsal Bridge], three thoracic segments, and eight abdominal segments), ΔHead: larvae missing one or more head segments with normal abdominal and thoracic segments, ΔAbdomen: larvae with variable defects in abdominal segments but normal head and thorax. If an embryo showed both abdominal defects and head defects, it was classified as ΔHead+Abd. The number of WTL, ΔHead, ΔHead+Abd and ΔAbdomen larvae were counted for each transgenic line, tabulated, and graphed as a percentage of the total number of embryos analyzed for that transgenic line.

For lines producing WTL larvae, we set up hatching assays to assess survival of these larvae to pupa stage and then adulthood. For hatching assays, females containing rescue constructs were allowed to lay embryos on fruit juice plates for 1 h and then over 100 eggs were picked and incubated at room temperature until pupae formed and adults eclosed. Pupae and adults were counted, and compared with the number of embryos tested for each experiment.

### Measuring *hb* Pbps

To measure pbps of *hb* anterior expression, stained embryos of appropriate ages were imaged at 200× on a Zeiss Axioskop. Briefly, coordinates were established for each embryo so that the *x*- and *y*-axes were tangential to the ventral and anterior sides, respectively. A–P positions were displayed as percent of embryo length (EL%) with the anterior pole as 100%. pbps were determined by visual estimation as the distance from the anterior tip to the most posterior position of *hb* anterior expression and these results were confirmed by ImageJ analyses (ImageJ, RRID: SCR_003070) (Schneider et al. 2012; Rueden et al. 2017).

Embryo images were loaded into ImageJ, and a Region of Interest (ROI) that was approximately 35% width of DV length from 95% to 60% (where 100% is most dorsal side) to analyze expression patterns was generated. The width of the ROI was kept constant when imaging all embryos, but the length was varied such that the length of the ROI spanned the length of the whole embryo. For each embryo, an intensity profile plot (intensity v. position along embryo length) was generated for the ROI. The midpoint of the curve that represents the edge of the boundary of expression of target gene was selected as the position of the boundary of gene expression. This numerical position was divided by the total embryo length to normalize the pbps by % EL, and pbps from individual embryos were averaged. 100% EL denotes the anterior tip, and 0% represents the posterior tip of the embryo.

### Measuring *eve* Patterns

To measure *eve* stripe patterns, stained embryos at nc14 were positioned as described above and imaged at 200×. For each embryo, an intensity profile plot (intensity vs. position along embryo length, where 100% denotes the anterior tip) was generated using ImageJ (ImageJ, RRID: SCR_003070) (Schneider et al. 2012; Rueden et al. 2017), and analyzed using our Embryo Analyzer tool, which serves to take ImageJ plots of fly embryos, and convert them to produce a single file of normalized intensities along the AP axis of n number embryos. Estimation graphics were generated from the distribution of % EL positions of *eve* 1 by using a web application, available at https://www.estimationstats.com (last accessed February 23, 2021) (Ho et al. 2019).

## Supplementary Material

Supplementary data are available at *Molecular Biology and Evolution* online.

## Supplementary Material

msab051_Supplementary_Data
