# Supplementary material for: Suboptimal Intermediates Underlie Evolution of the Bicoid Homeodomain
Source: Mol Biol Evol. 2021 Feb 18;38(6):2179–90. doi: 10.1093/molbev/msab051 (PMC8136501; doi:10.1093/molbev/msab051)
Supplement: msab051_Supplementary_Data [file msab051_supplementary_data.pdf]

Suboptimal intermediates underlie evolution of the Bicoid homeodomain

Supplementary Figures S1-S5

Pinar Onal, Himari Imaya Gunasinghe, Kristaley Yui Umezawa, Michael Zheng, Jia Ling, Tasmima Tazin, Leen Azeez, Anecine Dalmeus, Stephen Small*

New York University, Department of Biology, NY, USA
*Corresponding Author: pinar.onal@nyu.edu

Fig. S1

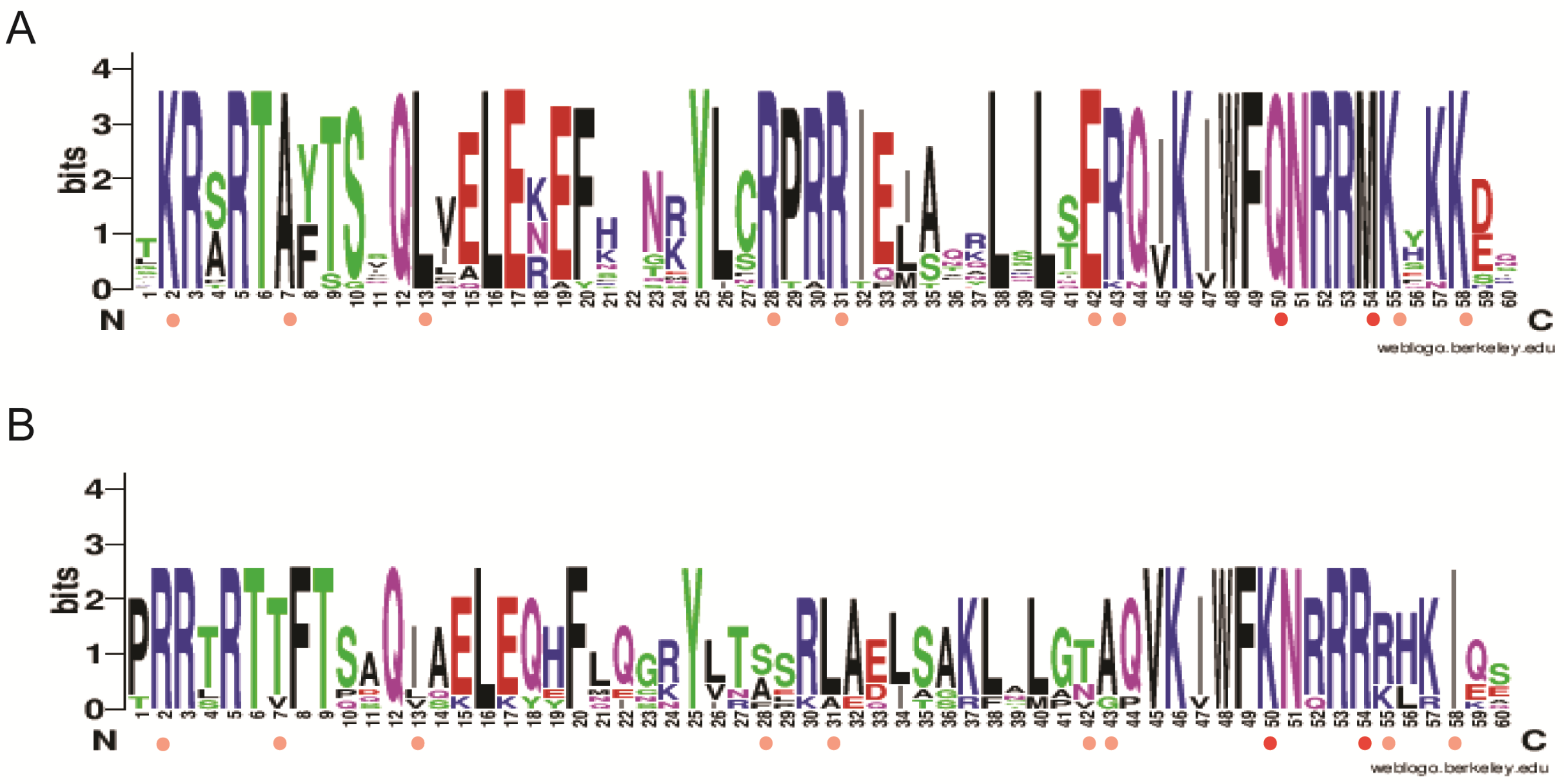

Fig.S1. *WebLogos of the insect Zen and Bcd HD sequences. Related to Fig. 1.*
A, B. The logos represent each position in the Zen (A) and Bcd (B) HD sequences by a stack of letters, with the height of each letter proportional to the frequency of the corresponding amino acid. The overall height of each stack is proportional to the sequence conservation, measured in bits, at that position. 11 diagnostic residues are designated with a dot underneath alignments. Already published Q50>K and M54>R substitutions are shown in darker red, the pale red ones are tested in this study.

Fig.S2

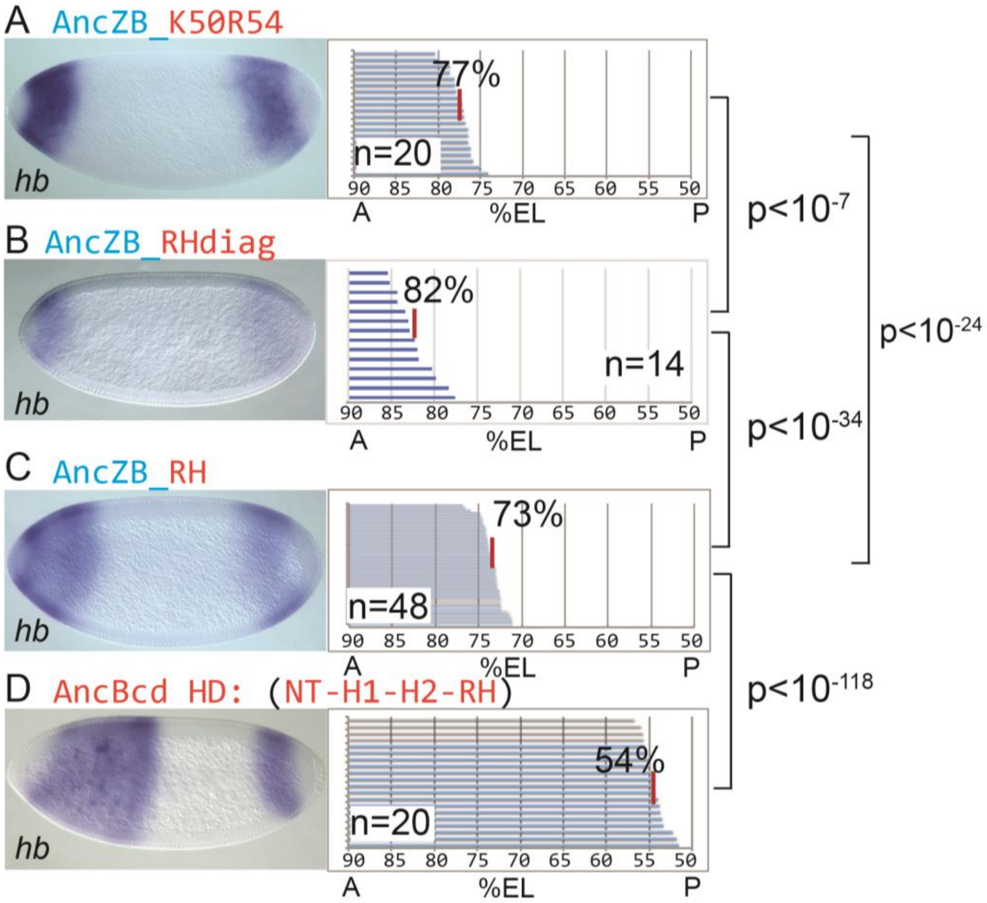

Fig. S2. *hb expression patterns and hb posterior boundary positions (pbps) of anterior hb patterns of stage 5 embryos from transgenic lines described in Fig. 2 and control AncBcd HD lines. Related to Fig. 2.* **A-D.** Left panels show representative nc14 embryos stained by *in situ* hybridization to detect *hb*. Same embryos as in Figure 2 are chosen as representative embryos. Panels to the right show the plot of *hb* posterior boundary positions (pbp) embryos (% EL; anterior tip = 100%) at nc14. Each horizontal line in each panel represents the anterior *hb* expression pattern in a single embryo, and the average pbp is denoted by a vertical red line. N numbers of embryos are used for each experiment. The most anterior tip is 100% EL (embryo length). Only 90 to 50 % EL is shown on the right panels. p-values calculated using student's t-test between the HD variants.

Fig. S3

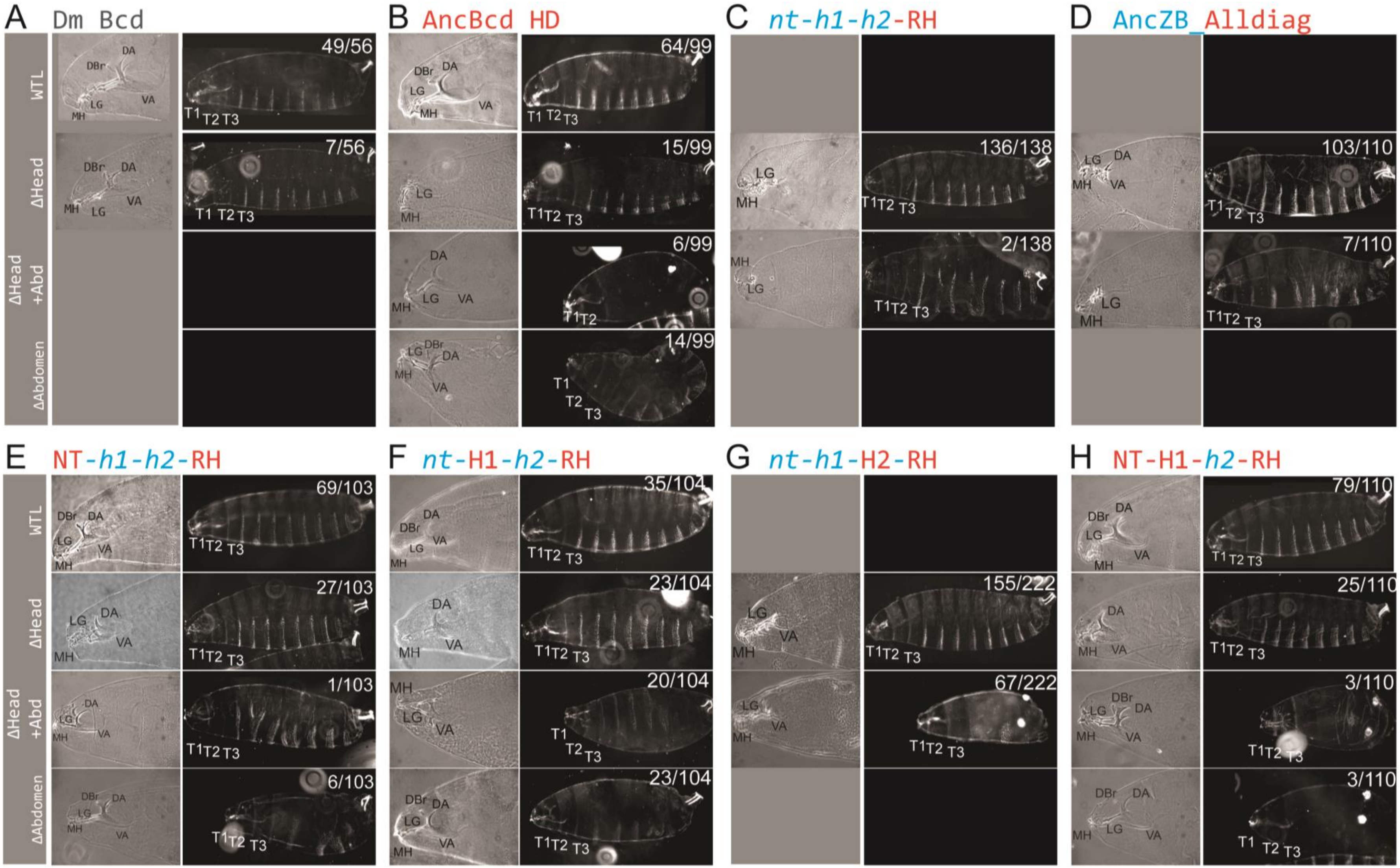

Fig. S3. *Phenotypic variation in transgenic D.melanogaster expressing ancestral proteins.* Related to Figs. 3 and 4. **A-H**. Representative of first instar larvae showing different degrees of morphological rescue upon expression of different chimeric ancestral HD. Morphologies of individual larvae were classified as described in the text and Methods. Left of each panel shows cuticle preparations of the head, and the right shows cuticle of the whole body of the transgenic larvae. Ratio given is the ratio of number of larvae counted for the category per the total larvae in one transgenic line.

Fig. S4

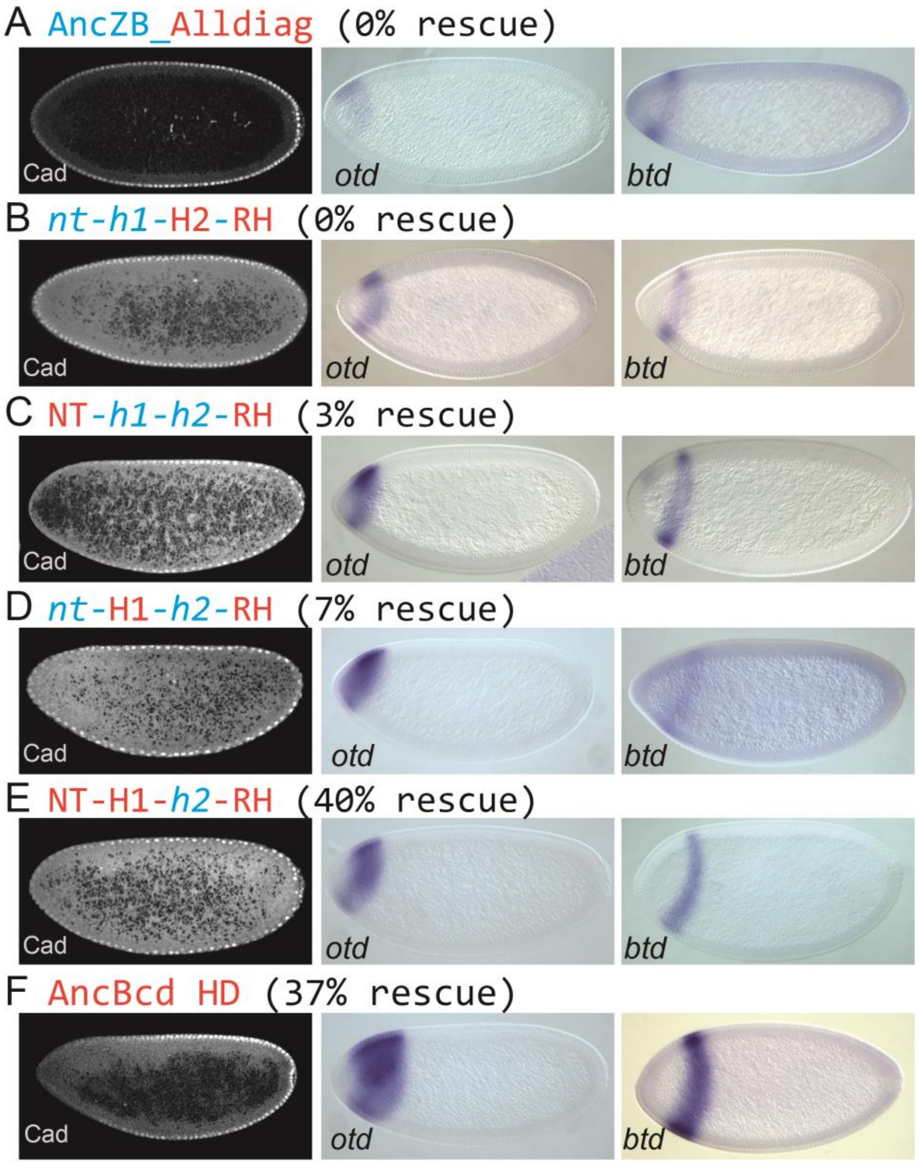

Fig. S4. *Transcriptional and translational activities of constructs that were described in Figs. 3 and 4.* **A-F.** Distribution of posterior determinant Caudal (Cad) protein and expression patterns of RNAs of head gap genes *otd (orthodenticle)* and *btd (button head)* of nc14 or younger embryos. Sagital views of nc11-13 embryos are presented for Cad immunostaining. Cad protein localized into the nuclei on the periphery is shown.

Fig. S5

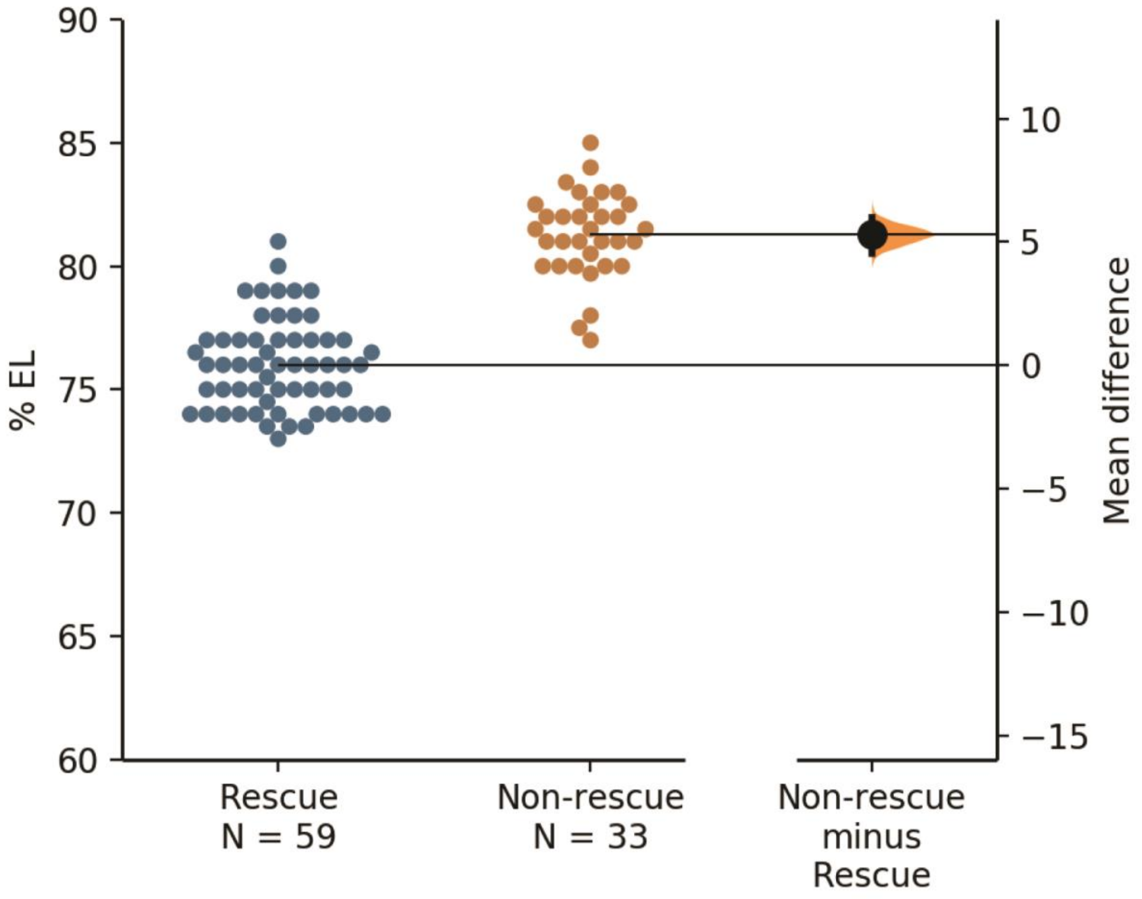

Fig. S5. *eve stripe 1 position distribution in rescuing vs non-rescuing lines. Related to Fig. 4.* The mean difference in *eve stripe 1* position between *rescuing* (NT-H1-*h2*-RH, **NT**-*h1*-*h2*-**RH**, *nt*-**H1**-*h2*-**RH**) vs non-rescuing (**NT**-**H1**-*h2*-**RH** and AncZB_AllDiag) is shown in the above Gardner-Altman estimation plots. Compared groups are plotted on the left axes; the mean difference is plotted on a floating axes on the right as a bootstrap sampling distribution. The mean difference is depicted as a dot; the 95% confidence interval is indicated by the ends of the vertical error bar.
